# Comment on Machine Learning–Based Prediction of Histopathological Classification in Colorectal Polyps

**DOI:** 10.5152/tjg.2026.25816

**Published:** 2026-02-20

**Authors:** Serdar Akca, Osman Cagin Buldukoglu, Galip Egemen Atar, Ayhan Hilmi Cekin

**Affiliations:** Department of Gastroenterology, Antalya Training and Research Hospital, Antalya, Türkiye

We read with great interest the article by Koker et al,^1^ “Machine learning–based prediction of histopathological classification in colorectal polyps,” published in the *Turkish Journal of Gastroenterology*.

Colorectal cancer is the third most prevalent cancer worldwide, and colorectal polyps can act as precursors for colorectal tumors.^2^^,^^3^ In recent years, several studies have explored machine learning (ML) models to predict the risk of colorectal polyps or adenomas prior to colonoscopy using noninvasive inputs such as demographic, lifestyle, and clinical data rather than imaging.^4^^,^^5^

In this context, we would like to emphasize that the authors’ attempt to apply ML models using demographic, clinical, and lifestyle variables to differentiate colorectal polyp types represents a valuable and innovative effort. Exploring non-imaging, pre-procedural data for histopathological risk estimation addresses an important unmet need in colorectal cancer screening research. In this respect, the study provides meaningful exploratory insights and contributes to the growing body of literature on explainable and accessible ML-based risk prediction models.

However, we would like to address an important aspect of the analysis and reporting that directly influences the interpretation of the Discussion and the proposed clinical applicability of the model.

In the Methods section, the authors stat that model performance was evaluated using a comprehensive set of metrics, including accuracy, sensitivity, specificity, positive and negative predictive values, F1-score, kappa statistic, and McNemar’s test. Despite this, the Results section primarily reports accuracy, sensitivity, and precision, whereas specificity and negative predictive value (NPV) are not clearly or consistently reported, particularly in a class-wise manner.

This point becomes particularly relevant in the Discussion, where the authors suggest that their ML model may support pre-procedural risk assessment beyond age-based screening strategies. For any model considered for such clinical applicability, the ability to reliably identify truly low-risk individuals is as critical as identifying high-risk patients. In this context, specificity and, most importantly, NPV are essential metrics, as they determine whether patients classified as low risk indeed have a low probability of clinically relevant polyps.

Without clear reporting of specificity and NPV, the risk of false negative predictions cannot be adequately assessed. If these metrics are low, patients with adenomatous polyps may be incorrectly classified as low risk, potentially limiting the clinical applicability of the model. Therefore, conclusions regarding the use of the model for supporting screening-related clinical decision making cannot be fully evaluated based on the reported results.

Moreover, given the evident class imbalance in the study population, accuracy and sensitivity alone may overestimate model performance. In imbalanced datasets, acceptable overall accuracy may coexist with a clinically relevant false-negative rate, further underscoring the importance of transparent reporting of specificity-related metrics.

In summary, while the study provides valuable exploratory data on the use of ML models incorporating demographic and lifestyle factors, the absence of clearly reported class-wise specificity and NPV limits the strength of conclusions regarding clinical applicability. Clarification and explicit reporting of these metrics would strengthen the interpretation of the findings and better inform their potential role in practice.

We thank the authors for their contribution and hope that this comment will be received in a constructive spirit.

## Data Availability

The data that support the findings of this study are available on request from the corresponding author.
